# Electragel for Advanced Static Charge Mitigation and Energy Harvesting

**DOI:** 10.1002/advs.202504600

**Published:** 2025-09-23

**Authors:** Irum Firdous, Muhammad Fahim, Kaixin Lin, Tsz Chung Ho, Yihao Zhu, Chi Yan Tso

**Affiliations:** ^1^ School of Energy and Environment City University of Hong Kong Hong Kong 00000 China

**Keywords:** electragel, electrostatic discharge protection, energy harvesting, nanogenerator, renewable energy

## Abstract

The heightened sensitivity of modern electronics has outpaced the effectiveness of traditional grounding, ionization, and environmental control methods, which necessitates the development of advanced technologies to reliably safeguard against electromagnetic interference and ensure consistent performance. This study introduces a material called Electragel, a highly transparent (92%) and adhesive (1.6 MPa shear strength) matrix, which can effectively scavenge static charges from stationary and dynamic sources to enable controlled dissipation or abundant electricity generation. This unique charge‐absorbing matrix can screen charges from a wide range of materials. Experiments showed that Electragel can reduce the voltage output of electrified surfaces from 1.9 to 0.2 V regardless of the geometry, spread area, or dissipation pattern of the static charges. With its high charge storage capacity, the Electragel matrix is leveraged to harness stored static charges as a source of ambient electricity and achieved maximal outputs of 15.5 W m^−^^2^. The Electragel system exhibited an output range of 20–632 mA m^−^^2^ under mechanical stimuli of 1–300 m s^−^^2^, which demonstrates its efficacy in static‐charge screening and mechanical endurance. The versatile static charge management of the Electragel system advances the understanding of charge dynamics and enables innovative energy and static control applications.

## Graphical abstract

Electragel is a transformative material that scavenges static charges for simultaneous dissipation and electricity generation, enabling dual‐purpose static control and energy harvesting across industries while shifting paradigms from mitigation to sustainable utilization of electrostatic energy.

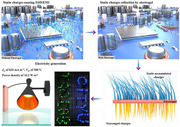

## 1 Introduction

Contact electrification is a ubiquitous phenomenon that develops a charge imbalance on two surfaces through friction, contact, or induction.^[^ ^1^, ^2^ ^]^ This elementary process of triboelectricity can be directly observed in daily life, e.g., lightning in thunderstorms, sandstorms, and volcanic eruptions. Contact electrification can lead to unwanted static charge accumulation, which can cause dust attraction, material adhesion, spark fire, electric shock, and interference with electronic devices.^[^ ^3^, ^4^, ^5^ ^]^ Empirical safety regulations have been established to prevent electric discharge hazards, including regulations about charge dissipation via ground connection, ionization, induction, incorporation of conductive materials, and humidity and temperature management. However, these mitigation systems suffer from surface contamination, inconsistent discharge standards, high costs, and reduced effectiveness in dusty environments. In addition to the increased sensitivity of modern electronics and potential electromagnetic interference, these issues underscore the need for enhanced technologies in static‐charge management.^[^ ^6^ ^]^

Recently developed self‐powered triboelectric energy harvesters (TEHs) effectively transform mechanical energy into electrical energy by combining contact electrification and electrostatic induction.^[^ ^7^ ^]^ This approach enables TEHs to neutralize static charges in real time and eliminates the need for external power sources and reduction in particle generation, which are typically associated with conventional ionizers.^[^ ^8^ ^]^ In addition, TEHs can operate in varied environments, including high‐dust or challenging conditions. They are designed to dissipate static charges through multiple configurations, accommodating contact, sliding interactions, and free‐floating systems, where direct electrical connections may not be feasible.^[^ ^9^, ^10^, ^11^, ^12^, ^13^ ^]^ This flexibility enhances their capability to manage static electricity across diverse applications and settings.^[^ ^14^, ^15^, ^16^ ^]^

Beyond kinetic stimuli, static charges can be generated through friction, induction, electrostatic polarization, and corona effect.^[^ ^4^, ^17^, ^18^ ^]^ TEHs can mitigate static‐charge accumulation from continuous kinetic stimuli.^[^ ^19^, ^20^ ^]^ They are less effective against static charges that are induced by friction or contact with non‐moving surfaces, and they cannot eliminate charges that arise from environmental factors such as humidity or electrostatic polarization, which can occur with no mechanical input. For example, static charges can accumulate on surfaces due to environmental conditions or through processes such as charging by induction, where a charged object affects the nearby neutral objects without direct contact.^[^ ^21^ ^]^ Since TEHs depend on motion to function, they also cannot actively neutralize static charges in stationary scenarios. Therefore, although TEHs offer a promising solution to manage certain types of static electricity, complementary approaches are necessary to effectively address the broader spectrum of electrification challenges in modern electronics.

This study presents a novel approach to lock static charges from both stationary and dynamic electrified sources in an Electragel matrix, which enables one to control the static dissipation or harness the abundant electricity. Electragel is a super‐adhesive, transparent, electrically conductive gel with a floating‐charge‐absorbing matrix, which facilitates charge screening from diverse classes of materials: phthalates, fluoropolymers, acrylics, metals, siloxanes, glasses, conductive electrodes, and even neodymium magnets without a direct electrical ground. De‐electrification dynamics experiments were conducted to study the accumulation of static charges from rolling, flowing, and rubbing contacts, which generated a maximal output of 1.9 V. This output was reduced to 0.2 V due to the induction of static charges into the Electragel, regardless of the geometry and area of the electrified surface. The electricity generation from tribopositive, tribonegative, conductive, and magnetic electrification layers was very stable, where the highest output was to 15.5 W m^−2^. The charge transfer patterns resulting from the induction of charges from these electrification layers to Electragel were studied using in situ impedance spectroscopy combined with triboelectric perturbation. The overall design comprised of an Electragel layer that adhered to the electrification surface and an Electragel connection to the collector. The single‐layer design had stable electricity generation and remained intact under extreme variations in mechanical inputs of 1–300 m s^−^^2^ and frequencies of 0.25–100 Hz over 14 000 s operational loops. With increasing acceleration rate to 300 m s^−2^, the current density increased from 20 to 632 mA m^−2^, which demonstrates the ability of Electragel to effectively screen static charges at high levels. The results demonstrate the effectiveness of Electragel in capturing and managing static charges from various electrified sources, advancing our understanding of charge dynamics, and opening new avenues for applications in energy generation and static electricity control.

## 2 Results and Discussion

### 2.1 Electragel for Static Charge Mitigation

Modern portable and wearable electronics, critical to advancing IoT and medical technologies, face escalating threats from electromagnetic interference (EMI) and electrostatic discharge (ESD), which degrade signal integrity, damage low‐voltage circuits, and compromise user safety. EMI—from wireless signals, power lines, or onboard components—introduces noise in sensitive sensors, while ESD events (e.g., human contact) deliver transient surges up to 15 kV, risking irreversible component failure. Current solutions like metallic shielding films, conductive foams, and transient voltage suppression (TVS) diodes struggle with trade‐offs: rigid shields limit device flexibility and transparency, polymer‐based additives sacrifice mechanical strength, and traditional grounding methods fail in miniaturized designs where space constraints exacerbate heat dissipation and parasitic capacitance. These limitations create unreliable protection in ultra‐thin, flexible, or transparent electronics, particularly in humid or dynamic environments.

**Scheme 1:**
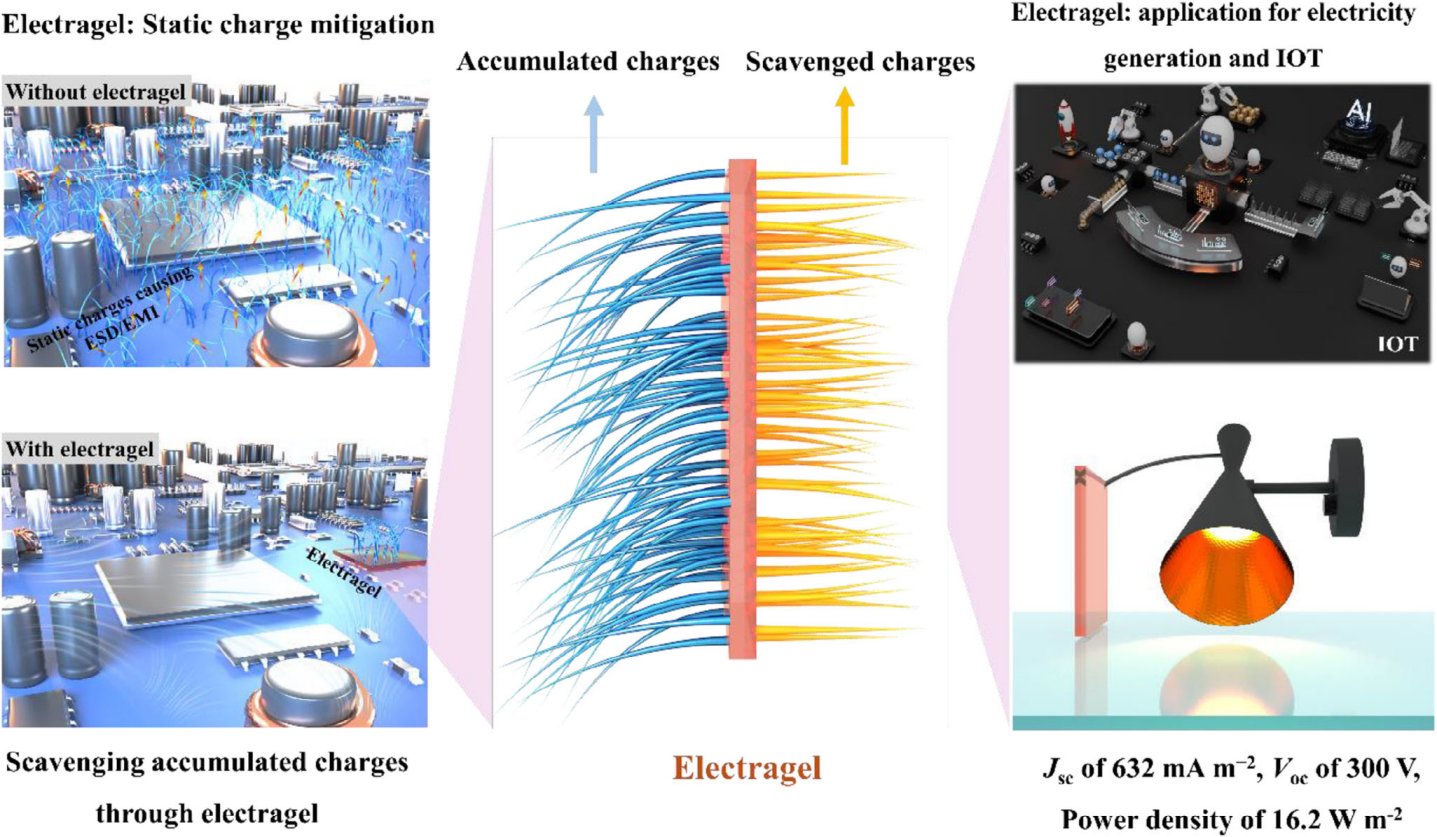
Schematic representation of Electragel applications in static charge mitigation and energy harvesting.

Electragel, a multifunctional ionogel‐based material, uniquely addresses these challenges while introducing transformative energy‐harvesting capabilities. With 92% transparency, 1.6 MPa shear strength, and 632 mA m^−^^2^ charge scavenging capacity, it neutralizes static charges in real time, replacing bulky TVS diodes, while its conductive ionic network reflects and dissipates EMI across frequencies critical for 5G and IoT. Beyond protection, Electragel's single‐electrode design harnesses scavenged electrification charges—such as triboelectric energy from motion or environmental friction—to generate electricity, converting otherwise disruptive static buildup into a power source for low‐energy sensors or auxiliary circuits, **Scheme** **1**. This dual functionality eliminates the need for separate shielding and energy‐harvesting components, a stark advantage over conventional materials. Its adhesive properties further streamline integration, serving as structural bonds in foldable devices or transparent coatings for displays without compromising aesthetics. By unifying EMI/ESD mitigation, mechanical resilience, and self‐sustaining energy recovery, Electragel transcends traditional trade‐offs, offering a scalable, multifunctional solution for ultra‐compact electronics where efficiency, durability, and sustainability are critical. The study will evaluate Electragel's effectiveness as a static charge mitigation material through non‐grounded coupling mechanisms by analyzing charge dissipation dynamics.

### 2.2 Electragel Non‐Grounded Couplings Mechanism

De‐electrification dynamics refers to the reduction or neutralization of static charge on surfaces to manage static electricity^[^ ^22^ ^]^ that arises from the imbalance of electric charges, which are often generated through friction, contact, or induction. When two charged surfaces of identical polarity come into contact, the electric field strength around these materials increases. If this strength exceeds the dielectric breakdown threshold of the surrounding, static charges dissipate into the surrounding environment via dielectric breakdown, which causes contact de‐electrification. The accumulated charges can be controlled to dissipate in air because gases have considerably lower dissipation barriers than liquids and solids.^[^ ^8^ ^]^ Therefore, all conventional de‐electrification methods focus on neutralizing static electricity and managing charge accumulation through two complementary strategies: 1) enhancing charge dissipation in air via ionization, induction, and humidity/temperature control, which optimize air's natural dielectric properties, and 2) directing charges to solids through grounding or the integration of conductive materials, bypassing air entirely by providing low‐resistance pathways.^[^ ^2^, ^4^ ^]^ This study introduces a novel approach to static charge dissipation using Electragel, a solid conductive matrix that scavenges accumulated charges from surfaces without requiring grounding or external conductive components. Unlike conventional air‐mediated dissipation, Electragel provides a direct, low‐barrier pathway for charge neutralization, enabling faster static decay rates than those achievable through atmospheric breakdown.

**Figure 1:**
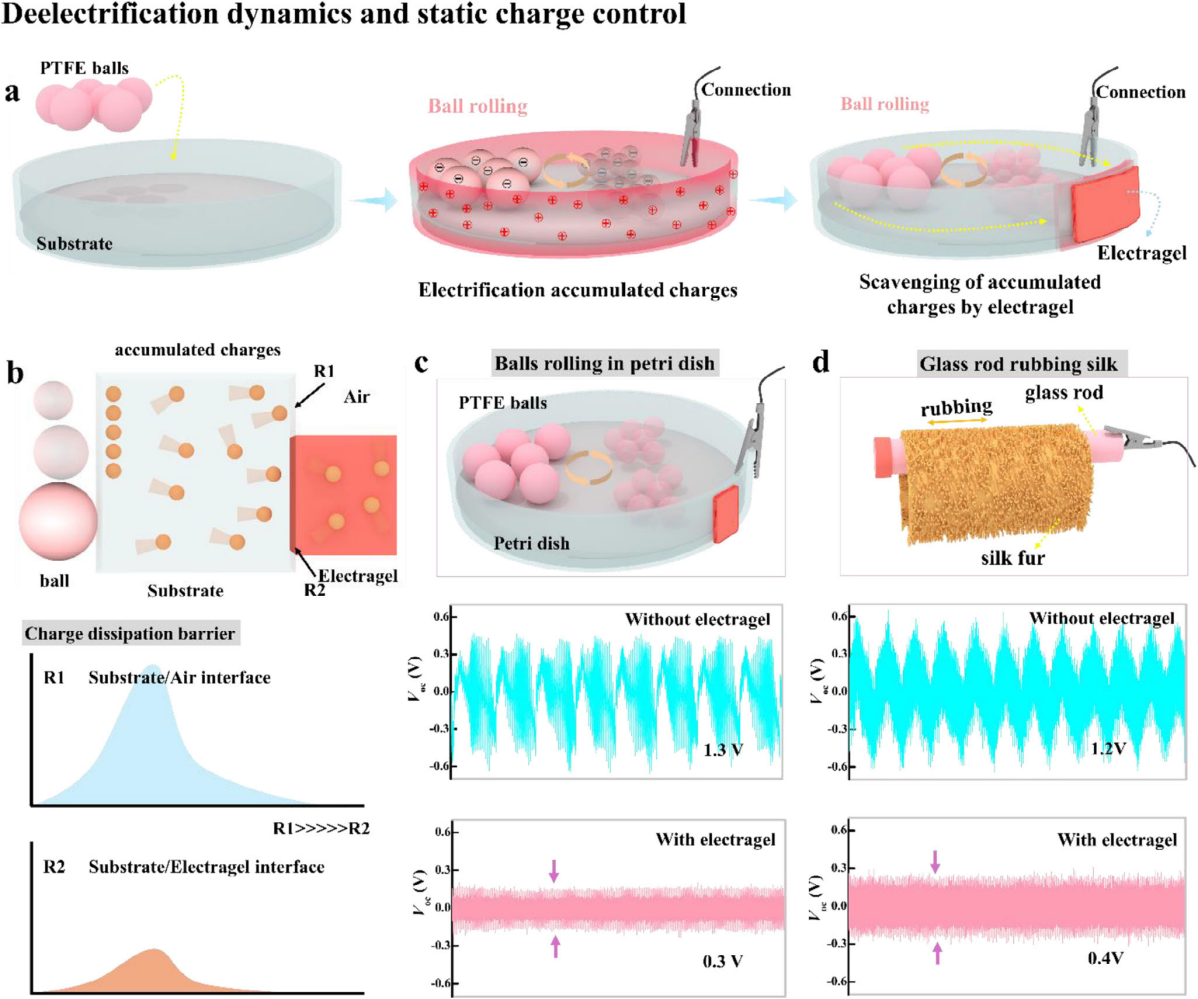
Deelectrification dynamics and static charge control by Electragel. a) Schematics of the contact electrification setup with PTFE balls rolling in a PET petri dish to generate static charges and dissipate to the attached Electragel; b) schematics of the static‐charge dissipation to Electragel; c,d) effect of the static charge dissipation to Electragel on the charge density of electrification surface in terms of output voltage.

The five cases were chosen to encompass key static generation mechanisms encountered in industrial systems: rolling friction (PTFE balls modeling granular transport), liquid–solid interactions (water flow in PP tubes mimicking pipelines), and triboelectric charging (glass/silk/leather representing machinery friction and human contact). This selection tests Electragel's effectiveness across diverse geometries (flat surfaces, curved tubes) and material interfaces (solid–solid, liquid–solid), demonstrating broad applicability for real‐world scenarios involving particulate handling, fluid dynamics, and mechanical systems. The de‐electrification was evaluated in terms of the decrease in output voltage from two dielectric surfaces that were susceptible to electrification due to continuous friction. Examples:

1. Polytetrafluoroethylene (PTFE) balls were allowed to continuously roll in a polyethylene terephthalate petri dish, which was connected to a source meter to measure the charge generated from the rolling PTFE balls (**Figure** **1**a). As the PTFE balls continuously rolled, the dish surface generated static charges, which resulted in a measurable output voltage. The static charges remained evenly distributed across the dish surface due to the high dissipation barrier of the air (Figure 1b). When a 1 cm^2^ Electragel was attached to one side of the dish, it effectively screened the accumulated charges due to their low dissipation barrier, which noticeably reduced the voltage output. Thus, the open‐circuit voltage of 1.3 V of the original dish decreased to 0.3 V when the dish was screened by the attached Electragel (Figure 1c). This superior voltage reduction capability of Electragel was further demonstrated through a comparative analysis against conventional hydrogel materials, as detailed in Note S3 (Supporting Information).
2. When PTFE balls were placed in the polypropylene (PP) centrifuge tube, they generated an output voltage of 1 V. This voltage decreased to 0.4 V when Electragel was attached to the narrow end of the tube (Figure S1a, Supporting Information).
3. A polypropylene (PP) centrifuge tube filled with water was moved back‐and‐forth continuously to generate static charges from the flow of the polar liquid over the PP surface. This motion produced a static charge of 1.9 V, which decreased to 0.14 V when Electragel was attached to the oblong end of the tube (Figure S1b, Supporting Information).
4. A 15‐cm‐long glass rod with one end connected to a source meter was rubbed with silk fur to produce a 1.3 V output, which was reduced to 0.5 V when Electragel was attached on the other end of the glass rod (Figure 1d).
5. The same glass rod produced an output voltage of 1.7 V when it was rubbed against a leather seat. This voltage decreased to 0.4 V when Electragel was connected to the opposite end of the rod (Figure S1c, Supporting Information).

Electragel achieves 60–93% voltage reduction versus conventional methods—grounding (≈95% reduction) requires physical earth connections, limiting insulated/portable systems, while ionization (85–99% via corona/radioactive sources) needs continuous power and maintenance.^[^ ^8^ ^]^ In contrast, Electragel operates without grounding or external power, enabling use in enclosed systems (e.g., vacuum chambers), mobile equipment, and explosive environments where conventional methods risk sparks. Though slightly less effective than optimal grounding, its non‐contact, material‐based mechanism suits flexible electronics, biomedical devices, and scenarios where traditional approaches are impractical or hazardous.

### 2.3 Electragel Fabrication and Properties

Full synthesis and characterization details are provided in Notes S1 and S2 (Supporting Information). A chemical oxidative free radical polymerization was adopted to design a network of tercopolymer, as detailed provided in Experimental Section. The fabrication of this anionic terpolymer adhesive is intentionally designed to leverage complementary chemical functionalities for robust adhesion in aqueous environments. Acrylic acid (AA) provides carboxyl groups (−COOH, pKa ≈ 4.2) that ionize to −COO^−^ in water, enabling hydrogen bonding and pH‐responsive adhesion, while acrylamide (AAm) contributes amine groups (−NH_2_​) that TEMED deprotonates to enhance cross‐linking reactivity and chain flexibility. AMPS introduces strongly ionized sulfonic acid groups (−SO_3_^−^​, pKa ≈ 6.9), ensuring hydration stability and electrostatic adhesion across diverse pH conditions. Sodium borate crosslinks these ionized functional groups (−COO^−^, −NH^−^, −SO_3_^−^​) via dynamic boron‐diol ester bonds, creating a reversible network that balances cohesive strength (through self‐healing crosslinks) and adhesive capacity (via free functional groups). The ammonium persulfate (APS)/TEMED initiator system generates sulphate radicals efficiently at ambient conditions, ensuring uniform polymerization. This synergistic design combines AA's wet adhesion, AAm's hydrogen bonding flexibility, AMPS's ionic resilience, and borate's dynamic cross‐linking to achieve a multifunctional adhesive optimized for applications where competing interactions are critical.

**Figure 2:**
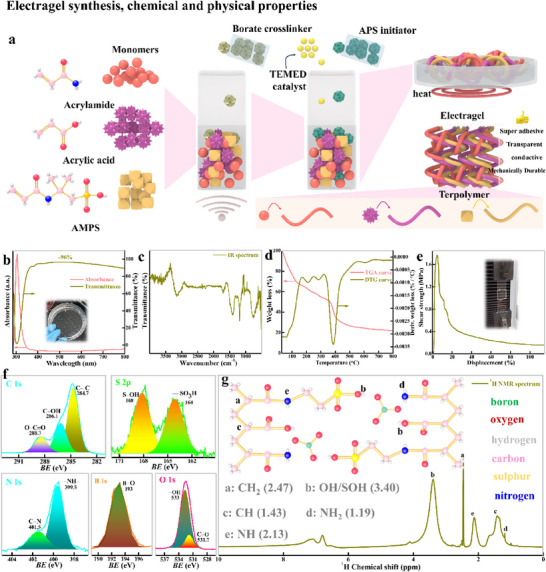
Synthetic protocol and molecular structure of Electragel. a) Schematics of the radical‐free polymerization to synthesize the tercopolymer Electragel. b) Optical transmittance/absorption spectra of electronic excitation, c) IR spectrum of the functional group arrangement, d) thermal decay profile, e) lap shear strength profile, f) and overall chemical environment of Electragel with bonding at the interacting valence orbitals. g) Distribution of hydrogen throughout the structure, as observed via the ^1^HNMR spectrum.

The molecular architecture of the terpolymer was rigorously characterized through spectroscopic techniques to verify structural integrity and functional group interactions. The tercopolymer was highly transparent (> 93%) in the range of 900–300 nm and revealed an absorption peak at 304 nm, which is associated with the *π–π* ^*^ transitions of the carbonyl groups^[^ ^23^ ^]^ in the amide functional groups of the AMPS block (**Figure** **2**b). The carbonyl functionalities of the carboxylic acid and amide groups are endorsed by significant bands at 1700 and 1674 cm^−1^ of the IR spectrum, respectively. The tercopolymer is hydrophilic, as indicated by the –NH and –OH group peak at 3200 cm^−1^. The peaks at 1160–1060 cm^−1^ are associated with the sulfonic group and indicate the ionic properties of tercopolymer. The band at 1258 cm^−1^ corresponds to B–O–H from sodium borate and confirms the cross‐linking and available slot for hydrogen bonding^[^ ^24^, ^25^ ^]^ (Figure 2c). The thermal profile indicates a 25% occluded water content, which was gradually eliminated from matrix till 100 °C; the absence of an abrupt decline peak below 100 °C indicates a high cross‐linking density^[^ ^26^ ^]^ (Figure 2d). The adhesion strength of the tercopolymer sandwiched between glass slabs demonstrates an impressive adhesion capability; it peaked at ≈1.6 MPa (Figure 2e; Movie S1, Supporting Information). This high adhesion value indicates a robust bond between ionogel and glass surfaces and is attributed to strong intermolecular interactions such as hydrogen bonding and electrostatic forces. Upon shear displacement, the bonds weakened with decreasing effective area.^[^ ^27^ ^]^ The XPS analysis (Figure 2f) further confirms characteristic binding energies of tercopolymer: aliphatic carbons (C 1s at 284.7 eV), sulfonic sulphur (S 2p at 168.0 eV), and amide/acrylic oxygen/nitrogen (O 1s at 531.5 eV, N 1s at 399.8 eV).^[^ ^28^ ^]^ The ^1^H NMR spectrum verifies monomer integration via distinct proton environments: AMPS methylene (2.47 ppm), acrylamide NH_2_ (1.19 ppm), and overlapping amide protons (2.13 ppm),^[^ ^29^ ^]^ Figure 2g and Note S2 (Supporting Information).

Overall, the Electragel acts as a floating charge‐absorbing matrix which functions by internally absorbing and neutralizing electrostatic charges through ion migration and dipole polarization within its bulk material, rather than shunting them to an external earth connection. The molecular‐level mechanism of this charge‐absorbing matrix is rooted in its tailored tercopolymer design, which enables both efficient charge sequestration (1.9 V) and controlled release (0.2 V residual). The network containing strongly ionized sulfonic acid groups (‐SO_3_^−^) from AMPS, provides a high density of mobile ions that migrate to screen any external electrostatic field. Simultaneously, the dynamic boron‐diol ester crosslinks and the inherent dipoles of carbonyl and amide groups from the acrylic acid and acrylamide units polarize in response to the electric field, creating localized internal dipoles that further stabilize incoming charges. For controlled dissipation, the release pathway is determined by the electrical configuration: when connected to a circuit, the sequestered charges drive a measurable current through the external load, while in an open configuration, the charges undergo gradual recombination via ion mobility and dielectric relaxation within the matrix itself, preventing sudden discharge.^[^ ^30^ ^]^ This combination of mobile ions for rapid charge capture and dynamic crosslinks for structural stability allows Electragel to function as an efficient floating charge reservoir, capable of managing electrostatic energy without requiring a ground connection.

### 2.4 Harvesting Electricity from the De‐Electrification Dynamics

**Figure 3:**
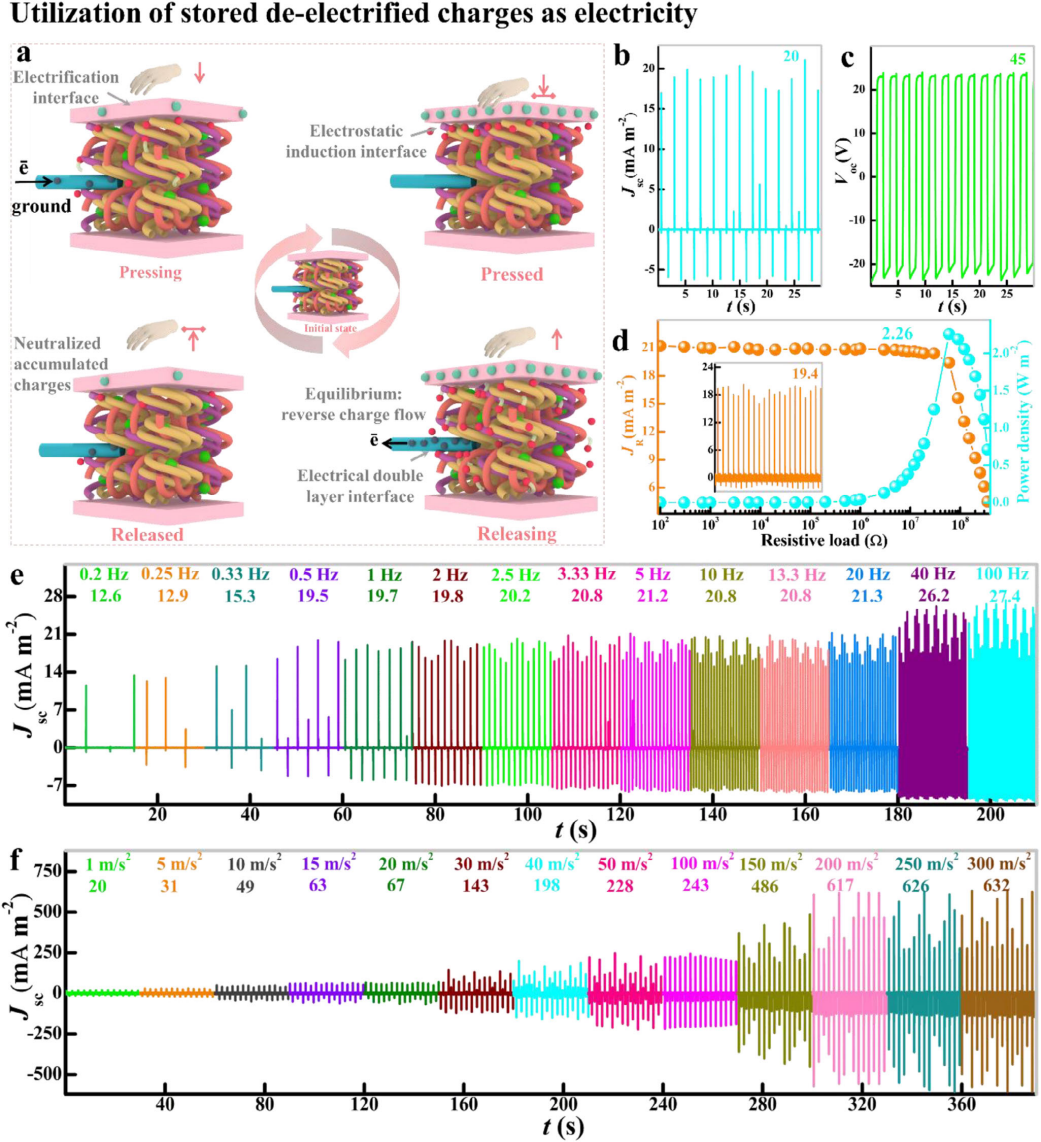
Electricity generation via the triboelectrification effect. a) Schematics of the working mechanism for energy‐harvesting charges from electrified solids via the Electragel induction mechanism. Electrical output from the continuous electrification: b) short‐circuit current density *J*_sc_; c) open‐circuit potential *V*_oc_, and d) current and power density profiles against the external load resistance. e) Impact of the enhanced kinetic energy on the device output current sensitivity at various frequencies of 0.2–100 Hz. f) Impact of the acceleration of the contact‐separation moving counter layer on the current output.

To harvest electricity from tough solid electrified objects, a single‐electrode energy harvester (t‐SEH) was designed, which consists of three layers: a negative electrification layer of brittle solid (n‐EL), an electrostatic induction layer (Electragel), and an electrical conductor (Ag lead) (**Figure** **3**a). The charge transfer cycle begins when any free‐floating solid positive electrification layer (p‐EL) contacts n‐EL, generates equal but opposite charges on both surfaces, and results in no electrical potential difference. When the surfaces separate, static charges on n‐EL induce an ion migration in Electragel and create a surplus of positive ions at the n‐EL/Electragel interface. Concurrently, an electric double layer (EDL) forms between Electragel and Ag, causes polarization and generates negative charges on Electragel and equal positive charges on Ag. Electrons flow from Ag to ground through an external load until the static charges on n‐EL are neutralized. This EDL exhibits high capacitance (≈0.1 F m^2^) and low voltage (≈10^−2^ V); this voltage below 1 V prevents electrochemical reactions. When the p‐EL is distanced from n‐EL, electrostatic equilibrium is maintained. However, when p‐EL approaches n‐EL again, this equilibrium is disrupted, which reverses the electron flow from ground to the Ag/Electragel interface through the external load. This cyclical approach and retreat of p‐EL generates alternating current signals by disrupting and restoring the electrostatic equilibrium.

When p‐EL directly contacts n‐EL, the quantity of short‐circuit charge (*Q*sc) is null with no open‐circuit voltage (*V*oc) across the interface. However, when p‐EL retrieves, Voc and Qsc become *V_oc_* =   − σ*A*/2*C_o_* and *Q_sc_* =   − σ*A*/2, respectively.^[^ ^31^, ^32^ ^]^ Here, σ is the density of electrostatic charges on the surface of n‐EL, C_o_ is the collective capacitance of the device, and A is the contact area between n‐EL and p‐EL. t‐SEH is an electrostatic system that exhibits inherent capacitive behavior that encompasses multiple capacitors. There are three capacitive layers at i) p‐EL versus n‐EL, ii) n‐EL versus Electragel induction layer, and iii) electrical double‐layer capacitance between Electragel induction layer versus Ag lead nodes.^[^ ^33^, ^34^ ^]^ The EDL that has developed at the releasing state of the charge transfer cycle determines the performance of the t‐SEH because charge induction is initiated on all interfaces at this stage.^[^ ^19^ ^]^

The t‐SEH connected to ground against PMMA as the positive counterpart shows a charge transfer of 26 nC (Figure S3, Supporting Information), a *V*oc of 45 V (Figure 3b), and a *J*sc of 20 mA m^−2^ (Figure 3c). The power density of t‐SEH was measured over a range of external resistances (10^2^–10^6^ Ω) and assessed using *P*  = *I* ^2^ *R*/*A*, where *I* is the output current over a cycle at resistance *R* for an active contact area *A*. The current output gradually decreased when load R was under 60 MΩ. With increasing resistance above 60 MΩ, the current rapidly decreased due to substantial charge usage by the resistor. The related power density considerably increased with the load resistance, and a peak power density of 2.26 W m^−2^ was obtained on the 60 MΩ resistor.

Single‐electrode electrification energy harvesting devices are commonly tested at low sequential operating frequency and acceleration rate to avoid interface detachment, fluid bulging, and device leakage. To assess the stability of the cross‐linked induction‐layer, the t‐SEH was subjected to extreme mechanical operational conditions of high contact separation frequencies of up to 100 Hz and acceleration/deceleration rates of up to 300 m s^−2^. The frequency variation of 0.2–100 Hz increased charge transfer *Q*_sc_ from 21.3 to 32.3 nC (Figure S4b, Supporting Information) with open‐circuit voltage *V*_oc_ of 37.2–57.2 V (Figure S4a, Supporting Information), and the corresponding short‐circuit current density *J*_sc_ was 12.6–27.4 mA m^−2^ (Figure 3e). When the frequency surged, the time period of the contact‐separation cycle decreased, and the charges on the electrification layer were nearly constant with minimal dissipation to the surrounding, which enhanced the performance.^[^ ^19^, ^35^, ^36^ ^]^ For mechanical endurance, t‐SEH was examined by varying the contact‐separation acceleration/deceleration rates from 1 to 300 m s^−2^. The t‐SEH had enhanced escalation in *J*_sc_ from 20 to 632 mA m^−2^ (Figure 3f) and in *V*_oc_ from 47 to 300 V (Figure S4c, Supporting Information). The t‐SEH charge accumulation and separation dynamics were optimized by the acceleration rate. The scaling of current density with acceleration arises from enhanced interfacial contact intimacy, which increases surface charge density (*σ*), coupled with a higher rate of charge separation (*dE*/*dt*), which amplifies displacement current and ion migration within the Electragel matrix. The t‐SEH device maintained full structural and functional integrity under extreme mechanical acceleration, with no observed bulging, leakage, or interfacial failure. This demonstrates both exceptional adhesion and robust electrical contact between the Electragel layer and the solid electrification surface. The device exhibits high power density along with superior mechanical endurance compared to all previously reported induction‐based energy harvesters employing solid, gel, or liquid intermediaries. Which confirms Electragel enhanced charge retention capability over conventional induction materials.^[^ ^34^, ^37^, ^38^, ^39^ ^]^ Further, the device exhibited no mechanical degradation—including cracking, delamination, or interfacial adhesion loss—after 14000 operational cycles at an acceleration/deceleration rate of 10 ms^−2^ (Figure S4d, Supporting Information). The t‐SEH maintains 100% performance under repeated mechanical stress, confirming its durable physical architecture and long‐term functional reliability. Furthermore, the environmental stability of Electragel under varying humidity and temperature conditions was confirmed, with the material maintaining high performance as detailed inNote S4 (Supporting Information).

**Figure 4:**
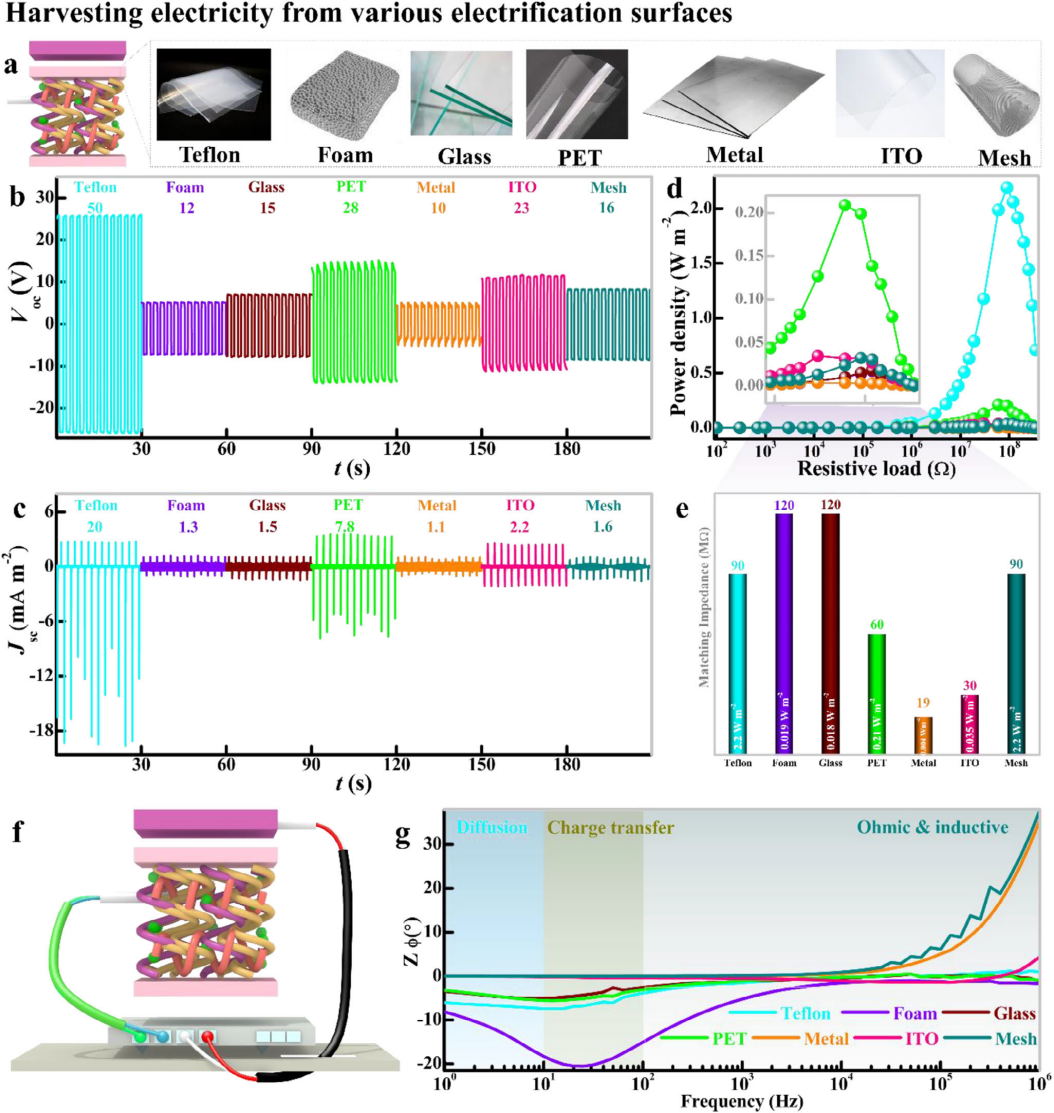
Electricity generation in the t‐SEH device conformation from various solid surfaces, a) as shown in the schematics of t‐SEH. b) Electrical output from t‐SEH as (b) *V*_oc_ and c) *J*_sc_ and their corresponding d) power density profile at the external load, considering the electricity generation capacity for each material as the e) power density at a specific matching impedance. f) Schematics of the setup for in situ EIS coupled with triboelectrification perturbation to verify the charge transfer mechanism of t‐SEH. Phase angle profile over the frequency range of 0.2–100 Hz.

Due to its strong fast‐forming interactive matrix, Electragel can adhere to various materials regardless of their morphology, surface roughness, and polarity with high shear strength (Figure S6, Supporting Information). However, to harvest electrification charges, both adhesion and higher induction of electrification charges than environmental dissipation are necessary. In this regard, seven tough solid electrification materials were used to fabricate t‐SEH: Teflon, foam, glass, PET, metal, ITO, and metallic mesh with Electragel as the induction layer and nitrile glove as the floating electrification layer (**Figure** **4**a). All devices showed very stable electrical outputs with *Q*_sc_ = 6–30 nC (Figure S7, Supporting Information), *V*_oc_ = 10–50 V (Figure 4b), and *J*_sc_ = 1.1–20 mA m^−2^ (Figure 4c). The power density of t‐SEH was measured over a range of external resistances (10^2^–10^6^ Ω) and assessed using *P*  = *I* ^2^ *R*/*A*, where *I* is the output current over a cycle at resistance *R* for an active contact area *A*. The current output gradually decreased when load R was below the matching resistance point. With further increase in resistance, a rapid decrease in current was observed due to substantial charge usage by the resistor (Figure S8, Supporting Information). The related power density considerably increased with the load resistance, and a peak power density at the matching resistance was obtained for various t‐SEH systems as depicted in Figures 4d,e. A significant divergence in power density across different material classes arises from distinct charge transport kinetics at the interface with Electragel. This variable charge harnessing mechanism for t‐SEH devices is further studied by in situ EIS measurements.

In measurements, a two‐electrode measurement setup was adopted, where one electrode from the induction layer (Electragel/Ag) was connected to the working electrode (WE) and working sense electrode (WS) cables, and another electrode from the floating electrification layer (nitril glove/Ag) was connected to the counter electrode (CE) and reference electrode (RE) cables. The potentiostatic testing mode was adopted for t‐SEH in the contact state. The triboelectrification perturbation capability of electrification layers guided the charge transfer pattern (Figure 4f). The phase angle profile of EIS over a wide range of frequencies manifested the charge transfer pattern in Figure 4g. There were three regions: 10^−2^–10^0^ Hz (low‐frequency diffusion region), 10^0^–10^2^ Hz (medium frequency, charge transfer region), and 10^2^–10^6^ Hz (high frequency, ohmic and inductive region). The activity in the low‐frequency region is attributed to the equivalent series capacitance and ion diffusion in the matrix. The medium‐frequency region shows an electrostatic displacement of charges or dipole delocalization, which is characteristic of a double layer. The high‐frequency region is attributed to the ohmic or inductive resistance that originates from the internal resistance of the electrode and Faradaic charge screening mechanism^[^ ^30^, ^40^, ^41^, ^42^ ^]^

For highly conductive layers, the EIS spectrum shows no dominant low‐frequency diffusion tail, indicating that charge transfer is not limited by ion diffusion. This is because the conductive surface acts as an extended electronic plane, enabling direct and rapid ionic‐electronic coupling at the Electragel interface. The nitrile glove (p‐EL), with its larger delocalized surface area, allows charges to intercalate more effectively into the counter electrode (CE). This process saturates the CE's capacity, thereby reducing the working electrode's (WE, Electragel/Ag) ability to induce further charge. Consequently, the charges around the WE are rapidly re‐neutralized within the Electragel matrix itself via ion recombination. This mechanism yields high current outputs due to fast kinetics but can limit maximum charge accumulation due to this efficient, instantaneous recombination.

In contrast, EIS spectra for dielectric layers exhibit a pronounced low‐frequency diffusion pattern, signifying that charge transfer is governed by field‐driven ion migration and dipole relaxation within the dielectric's bulk. The kinetics follow the order Foam > Teflon > PET > Glass, directly correlating with material porosity and molecular mobility. The open structure of foam facilitates electrodiffusion and greater interfacial ion penetration, increasing the effective chargeable interface. Teflon, despite its low surface energy, allows some dipole coupling. Dense, rigid dielectrics like glass show the most restricted ion mobility. Here, charges are not instantly neutralized at the interface. Instead, they accumulate, creating a sustained potential difference that drives higher voltage outputs. The high negative phase angle confirms a capacitive‐dominated process, where charges are stored at the interface rather than instantly recombined.

Overall, Electragel universal charge‐scavenging capability stems from its dual mechanistic adaptability: it employs direct ionic‐electronic coupling for conductors and field‐induced polarization for dielectrics. This versatility, enabled by its high ionic mobility and abundant polar groups, ensures effective electrostatic mitigation across diverse material interfaces, despite variations in power output due to substrate‐dependent kinetics.

**Figure 5:**
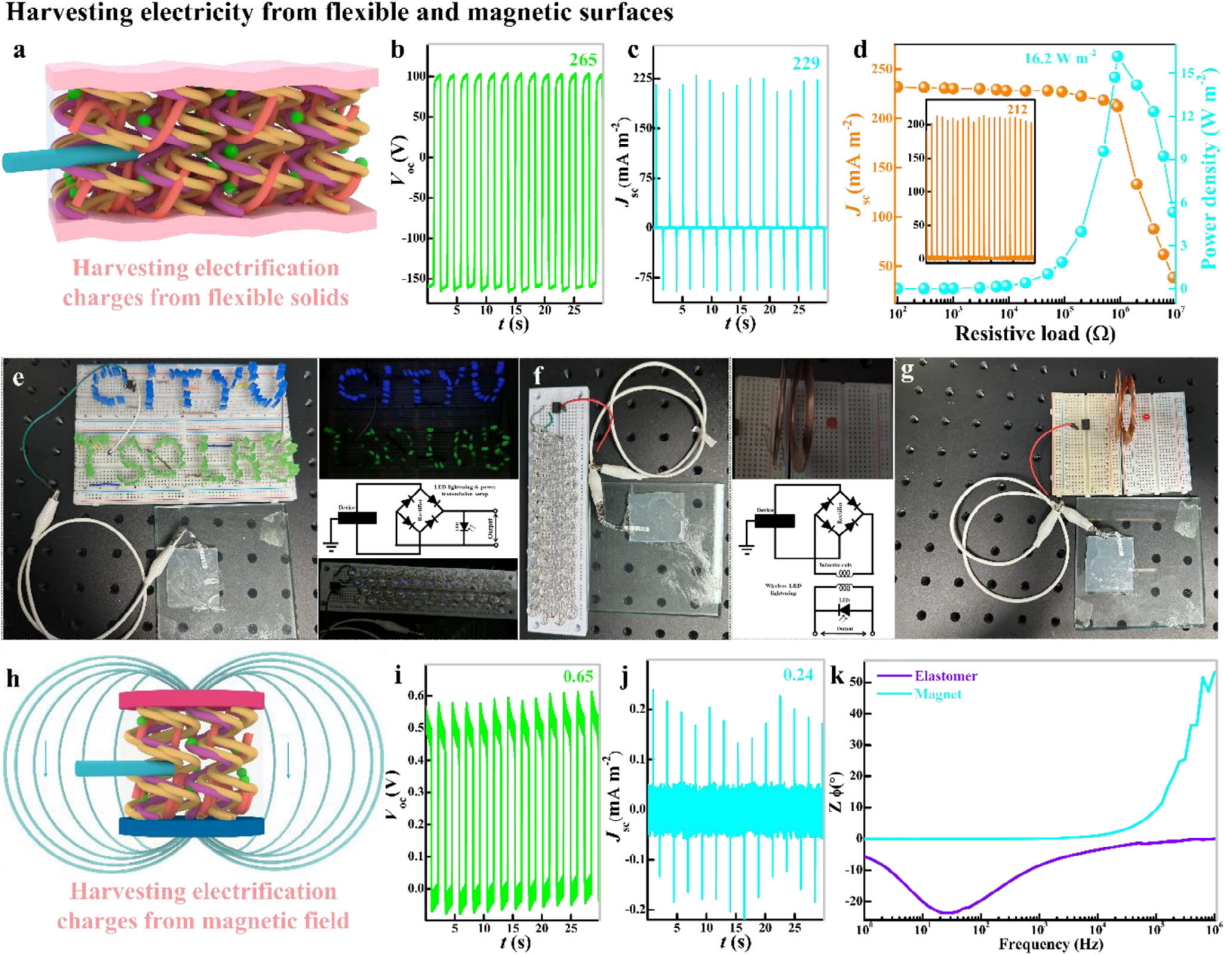
t‐SEH harvesting accumulated charges from flexible and magnetic substrates. a) Schematics of t‐SEH with flexible solids; electrical output from the b) open‐circuit potential *V*_oc_, and c) short‐circuit current density *J*_sc_; d) current and power density profiles against an external load resistance (10^2^–10^6^ Ω). A 9 cm^2^ t‐SEH with an AC output connected to LEDs via at the other end of bridge rectifier was used as a power source to light e) low‐power LEDs (0.06 W each) and f) high‐power LEDs (0.5 W each). g) Due to the high‐power density, the t‐SEH power transmission was evaluated by lighting LEDs via wireless connection of two inductive coils placed at a 1 cm distance. h) Harvesting accumulated charges from the magnetic field, which originated from neodymium magnet discs and their output performance i,j). k) Comparison of the charge transfer mechanism bode phaser profile.

To extend the applicability of Electragel, t‐SEH based on an elastomeric electrification layer was fabricated (**Figure** **5**a) and yielded a very stable electrical output with *Q*_sc_ = 124 nC (Figure S9, Supporting Information), *V*_oc_ = 265 V (Figure 5b), and *J*_sc_ = 229 mA m^−2^ (Figure 5c). The output current density was measured over a range of external resistances (Figure 5d). It gradually decreased when the load was below the matching resistance point, reached 212 mA m^−2^ as shown in the inset of Figure 5d, and had a peak power density of 16.2 W m^−2^ at 0.9 MΩ. The elastomer‐based t‐SEH was explored as a power source by converting its AC output to DC via a bridge rectifier, a connection to a load of low‐power LEDs (0.06 W each) and high‐power LEDs (0.5 W each) as shown in Figure 5e,f, and the electric circuit of the setup. The LEDs were lit and dimmed in a consecutive contact‐separation cycle endorsing (de)electrification process (Movie S2, Supporting Information). Furthermore, the wireless power transmission was demonstrated via a simple circuit setup with two inductive coils (inductance: 50 µH; diameter: 52 mm): one coil connected to t‐SEH via the bridge rectifier, and the other coil connected to the source meter or LED as shown in Figure 5g.^[^ ^43^, ^44^ ^]^ Due to the high power density and low internal resistance, the LED was lit when the t‐SEH was tapped as shown in Movie S3 (Supporting Information).

Neodymium magnets are widely used in renewable energy technologies, particularly electric vehicles (EVs), where it significantly enhances EV efficiency by generating powerful magnetic fields for propulsion, converting the regenerative braking kinetic energy into electrical energy, optimizing the battery heat management, and facilitating a lightweight infrastructure. However, the strength of the magnetic field decreases with time due to the continuous spontaneous electrification and applied‐current‐induced electrification, which results in heating, an eddy current in the magnet, and demagnetization.^[^ ^45^, ^46^, ^47^ ^]^ To maintain the magnetic strength, the screening of electrostatic charges from the neodymium disc was tested in the t‐SEH conformation (Figure 5h). t‐SEH generated a very stable electrification‐induced output with *Q*_sc_ of 0.30 nC (Figure S10, Supporting Information), *V*_oc_ of 0.65 V (Figure 5i), and *J*_sc_ of 0.24 mA m^−2^ (Figure 5j). The protective effect of Electragel against demagnetization was quantified by assessing magnetic flux stability under mechanical and environmental stress, with full experimental details provided in Note S5, Movies S4 and S5 (Supporting Information). Unlike the mass diffusion or charge transfer kinetics of elastomers, the charges from the magnetic layer were screened via the intrinsic resistance of the induction layer versus the magnetic layer (Figure 5k), which endorsed the capability of the hydrogel to neutralize the eddy current and prevent demagnetization.

## 3 Conclusion

In conclusion, this study presented a novel approach using Electragel to effectively capture and manage static charges from stationary and dynamic electrified sources. The findings reveal significant voltage reductions: the output generated from static charge accumulation decreased from 1.9 to 0.2 V when Electragel was used, which demonstrates its capability to mitigate unwanted electrical discharges. Additionally, the electricity generation from various electrification layers was stable with a maximum output of 15.5 W m^−^^2^, whereas the current density dramatically increased from 20 to 632 mA m^−^^2^ when the acceleration rate reached 300 m s^−^^2^. These results highlight the remarkable potential of Electragel for diverse applications, including energy‐harvesting systems that convert mechanical energy into electrical energy, improved electrostatic discharge protection for sensitive electronic devices, and effective static electricity control in industrial processes. The ability of Electragel to neutralize static charges without requiring grounding or external power sources enhances its applicability in challenging environments. Overall, this research advances our understanding of the charge dynamics and underscores the importance of innovative materials like Electragel in addressing the challenges posed by static electricity in modern technology.

## Conflict of Interest

The authors declare no conflict of interest.

## Supporting information



Supporting Information

Supplementary Movie 1

Supplementary Movie 2

Supplementary Movie 3

Supplementary Movie 4

Supplementary Movie 5

## Data Availability

The data that support the findings of this study are available in the supplementary material of this article.

## References

[1] M. Kaponig , A. Mölleken , H. Nienhaus , R. Möller , Sci. Adv. 2021, 7, abg7595. 10.1126/sciadv.abg7595 PMC8153716 34039611

[2] Z. L. Wang , A. C. Wang , Mater. Today 2019, 30, 34.

[3] Y. I. Sobolev , W. Adamkiewicz , M. Siek , B. A. Grzybowski , Nat. Phys. 2022, 18, 1347.

[4] L. B. Schein , G. S. P. Castle , D. J. Lacks , in Wiley Encyclopedia of Electrical and Electronics Engineering, John Wiley & Sons, New York, USA 1999, pp. 1–14.

[5] S. Pollastri , E. Rossi , C. Bonadonna , Sci. Adv. 2022, 8, abo7380. 10.1126/sciadv.abo7380 PMC9674278 36399553

[6] P. Molinié , J. Electrostat. 2024, 129, 103930.

[7] F. R. Fan , Z. Q. Tian , Z. L. Wang , Nano Energy 2012, 1, 328.

[8] C. K. Ao , Y. Jiang , L. Zhang , C. Yan , J. Ma , C. Liu , Y. Jiang , W. Zhang , S. Soh , J. Mater. Chem. A 2022, 10, 19572.

[9] L. He , Y. Gao , D. Liu , Y. Hu , J. Shi , J. Zhang , X. Li , B. Jin , B. Zhang , Z. L. Wang , J. Wang , Sci. Adv. 2024, 10, ado5362. 10.1126/sciadv.ado5362 PMC11168474 38865464

[10] S. Gong , K. Li , J. Sun , J. Chen , H. Guo , Joule 2025, 9, 101763.

[11] J. M. Almardi , X. Bo , J. Shi , W. Li , F. Liu , I. Firdous , W. A. Daoud , J. Mater. Chem. A 2025, 13, 8435.

[12] R. Liu , Z. L. Wang , K. Fukuda , T. Someya , Nat. Rev. Mater. 2022, 7, 870.

[13] K. H. Lim , Y. Sun , W. C. Lim , S. Soh , J. Am. Chem. Soc. 2020, 142, 21004. 33284628 10.1021/jacs.0c06000

[14] Y. Yu , H. Li , X. Zhang , Q. Gao , B. Yang , Z. L. Wang , T. Cheng , Joule 2024, 8, 1855.

[15] Z. Wang , W. Liu , W. He , H. Guo , L. Long , Y. Xi , X. Wang , A. Liu , C. Hu , Joule 2021, 5, 441.

[16] J. Zhang , X. Wang , L. Zhang , S. Lin , S. Ciampi , Z. L. Wang , J. Am. Chem. Soc. 2024, 146, 6125. 38323980 10.1021/jacs.3c13674 PMC10921404

[17] J. Li , Y. Xia , X. Song , B. Chen , R. N. Zare , Proc. Natl. Acad. Sci. 2024, 121, 2318408121. 10.1073/pnas.2318408121 PMC10823170 38232282

[18] J. Kim , H. Ryu , J. H. Lee , U. Khan , S. S. Kwak , H.‐J. Yoon , S.‐W. Kim , Adv. Energy Mater. 2020, 10, 1903524.

[19] I. Firdous , M. Fahim , F. Mushtaq , W. A. Daoud , Nano Energy 2023, 116, 108817.

[20] D. Liu , X. Yin , H. Guo , L. Zhou , X. Li , C. Zhang , J. Wang , Z. L. Wang , Sci. Adv. 2019, 5, aav6437. 10.1126/sciadv.aav6437 PMC6450689 30972365

[21] I. Y. Suh , Z.‐Y. Huo , J.‐H. Jung , D. Kang , D.‐M. Lee , Y.‐J. Kim , B. Kim , J. Jeon , P. Zhao , J. Shin , S. M. Kim , S.‐W. Kim , Sci. Adv. 2024, 10, adl5067. 10.1126/sciadv.adl5067 PMC11067992 38701201

[22] S. Soh , S. W. Kwok , H. Liu , G. M. Whitesides , J. Am. Chem. Soc. 2012, 134, 20151. 23153329 10.1021/ja309268n

[23] E. G. M. van Dijk , et al., J Geophys Res Space Phys. 2009, 114, A00A10.

[24] R. M. Silverstein , G. C. Bassler , J. Chem. Educ. 1962, 39, 546.

[25] H. Günzler , H. U. Gremlich , IR Spectroscopy: An Introduction, Wiley‐VCH, Weinheim 2002.

[26] D. Mecerreyes , Prog. Polym. Sci. 2011, 36, 1629.

[27] A. Pizzi , K. L. Mittal Handbook of adhesive technology, CRC press, Boca Raton, Florida, USA 2017.

[28] J. F. Watts , J. Wolstenholme An introduction to surface analysis by XPS and AES, John Wiley & Sons, Hoboken, New Jersey, USA 2019.

[29] W. Kemp Organic spectroscopy, Bloomsbury Publishing, London 2017.

[30] I. Firdous , M. Fahim , R. Ye , J. Sik Chun Lo , C. Sze Ki Lin , W. A. Daoud , J. Mater. Chem. A 2025, 13, 22718.

[31] S. Niu , Z. L. Wang , Nano Energy 2015, 14, 161.

[32] S. Niu , Y. Liu , S. Wang , L. Lin , Y. S. Zhou , Y. Hu , Z. L. Wang , Adv. Funct. Mater. 2014, 24, 3332.

[33] J. Y. Sun , C. Keplinger , G. M. Whitesides , Z. I. Suo , Adv. Mater. 2014, 26, 7608. 25355528 10.1002/adma.201403441

[34] X. Pu , M. Liu , X. Chen , J. Sun , C. Du , Y. Zhang , J. Zhai , W. Hu , Z. L. Wang , Sci. Adv. 2017, 3, 1700015. 10.1126/sciadv.1700015 PMC5451198 28580425

[35] Z. Li , C. Yang , Q. Zhang , G. Chen , J. Xu , Y. Peng , H. Guo , Research 2023, 6, 0237. 37746657 10.34133/research.0237 PMC10516179

[36] A. Ahmed , I. Hassan , A. S. Helal , V. Sencadas , A. Radhi , C. K. Jeong , M. F. El‐Kady , iScience 2020, 23, 101286. 32622264 10.1016/j.isci.2020.101286 PMC7334414

[37] P. K. Jani , K. Yadav , M. Derkaloustian , H. Koerner , C. Dhong , S. A. Khan , L. C. Hsiao , Sci. Adv. 2025, 11, adr4088. 10.1126/sciadv.adr4088 PMC11734710 39813335

[38] Y. Wang , H. Wu , L. Xu , H. Zhang , Y. Yang , Z. L. Wang , Sci. Adv. 2020, 6, abb9083. 10.1126/sciadv.abb9083 PMC7438107 32875115

[39] C. Dong , A. Leber , D. Yan , H. Banerjee , S. Laperrousaz , T. D. Gupta , S. Shadman , P. M. Reis , F. Sorin , Sci. Adv. 2022, 8, abo0869. 10.1126/sciadv.abo0869 PMC9651858 36367937

[40] A. C. Lazanas , M. I. Prodromidis , ACS Meas. Sci. Au 2023, 3, 162. 37360038 10.1021/acsmeasuresciau.2c00070 PMC10288619

[41] N. O. Laschuk , E. B. Easton , O. V. Zenkina , RSC Adv. 2021, 11, 27925. 35480766 10.1039/d1ra03785d PMC9038008

[42] D. Qu , W. Ji , H. Qu , Commun. Mater. 2022, 3, 61.

[43] Y. Chen , Y. Cheng , Y. Jie , X. Cao , N. Wang , Z. L. Wang , Energy Environ. Sci. 2019, 12, 2678.

[44] H. Wu , S. Wang , Z. Wang , Y. Zi , Nat. Commun. 2021, 12, 5470. 34526498 10.1038/s41467-021-25753-7 PMC8443631

[45] X. Tang , J. Li , H. Sepehri‐Amin , A. Bolyachkin , A. Martin‐Cid , S. Kobayashi , Y. Kotani , M. Suzuki , A. Terasawa , Y. Gohda , T. Ohkubo , T. Nakamura , K. Hono , NPG Asia Mater. 2023, 15, 50.

[46] Q. Liang , Q. Ma , H. Wu , R. Lai , Y. Zhang , P. Liu , T. Qi , Sci. Rep. 2024, 14, 28822. 39572633 10.1038/s41598-024-79435-7 PMC11582802

[47] K. Wang , C. Yi , C. Liu , X. Hu , S. Chuang , X. Gong , Sci. Rep. 2015, 5, 9265. 25783755 10.1038/srep09265 PMC4363869

