# Supplementary material for: Electragel for Advanced Static Charge Mitigation and Energy Harvesting
Source: Adv Sci (Weinh). 2025 Sep 23;12(46):e04600. doi: 10.1002/advs.202504600 (PMC12697782; doi:10.1002/advs.202504600)
Supplement: Supplementary file 1 — Supporting Information [file ADVS-12-e04600-s001.docx]

**Supporting Information**

**Electragel for Advanced Static Charge Mitigation and Energy Harvesting**

**Irum Firdous, Muhammad Fahim, Kaixin Lin, Tsz Chung Ho, Yihao Zhu, Chi Yan Tso**

**Supplementary Note 1**

**Methods**

**Materials**

2-acrylamido-2-methyl-1-propane sulfonic acid (AAMPSA, 98%, Aladdin) and acrylic acid (AA, 99%, Aladdin) were employed as received anionic monomers. Acrylamide (Aam, 99%), Tetramethyl ethylenediamine (TEMED, 99%), and Ammonium Persulfate (APS, 98%) were procured from Aladdin. Sodium borate anhydrous was obtained from Macklin. Throughout the experimental investigation, deionized water served as the solvent.

**Synthesis of Electragel**

A tercopolymer Electragel was synthesized utilizing the free-radical polymerization technique. The monomer stock solution was prepared by dissolving 1.43 g of AA, 0.75 g of Aam, and 0.15 g of AMPS in 8 mL of deionized water. Different amounts of sodium borate anhydrous (0.05 g, 0.1 g, 0.15 g, or 0.2 g) were added as the crosslinker. The solution was sonicated for 15 minutes to ensure homogeneity. Subsequently, 20 μL of accelerator TEMED was introduced, followed by heating the solution at 70 °C on a hotplate for 2 minutes. Afterward, 2 mL of oxidant APS (5%, 0.16 g) was added while stirring at 700 rpm. The reaction mixture was covered with aluminium foil and heated at 70 °C for 30 minutes.

**Material Characterization**

The absorbance and transmittance of the film and device were measured with Shimadzu 2600 UV–vis spectrophotometer equipped with a 60 mm integrating sphere. The chemical structure and composition of the samples was analysed using infrared spectrophotometer (Shimadzu, Japan) at attenuated total reflectance (ATR) mode with a resolution of 4 cm^-1^ and scanning range of 400-4000 cm^-1^. Gamry reference 3000 ZRA potentiostat/Galvanostat was used to perform electrochemical impedance spectroscopy (EIS). X-ray photoelectron spectroscopy (XPS, PHI Model 5802) was employed to investigate the chemical composition of Electragel. The water content was removed from the hydrogel before testing using freeze drying chamber connected to vacuum. The lap shear test was conducted by adhering Electragel to two pieces of similar material clamped at an area of 10 × 10 mm^2^. A stretching force rate of 50 mm min^−1^ is applied parallel to adhesive bond until failure occur to find the maximum shear force sustained by the bond using mechanical tester (Instron 34SC-05, USA).

**Fabrication of all tough solid single electrode energy harvester (t-SEH)**

Hard all solid-state electrification energy harvester is fabricated by placing 0.3 mL viscous solution of Electragel on a 20 × 20 mm^2^ solid substrate and sandwiched by another layer of the same substrate with dimensions. The device was left for 5 minutes to develop strong crosslinking with high adhesion to both layers. The lower substrate has a conductive Cu-Ni mesh tape for the collection of electrification charges. For flexible t-SEH, polysiloxane elastomer (SR, ecoflex 00-50) with 1:1 weight ratio of base and cure were mixed and spread on the 2 cm × 2 cm × 1 mm template (with 1cm^2^ active area) to dry at room temperature. Each 1cm^2^ unit with concave mold is spun with uncured SR at the edges and 0.3 mL viscous solution of Electragel was placed in the center. A second SR mold is placed on the top, the device was dried overnight to fully cure the inside and outside SR. A metallic lead for charge collection was inserted from the side until reached the induction layer. The device has final dimensions of 20 × 20 × 1 mm^3^ with an enclosed 10 × 10 mm^2^ electrically active induction layer. A screw gauge was used to measure the thickness of the device.

**Electrical Characterization**

The output measurement was conducted in a meticulously designed aluminium chamber, incorporating a linear motor (LinMot), facilitated with the application of periodic mechanical compression input of 8 N, operating at a velocity of 1 ms^−1^ and an acceleration/deceleration of 1 m s^−2^. The electrical output of the t-SEH was evaluated utilizing a Keithley 6514 electrometer (input resistance = 200 teraohms) for measuring transferred charges, output voltage, and a low-noise current preamplifier (Stanford Research Systems, model SR570, impedance = 4 ohms) for measuring short circuit current. The software platform employed for data acquisition and analysis was developed using LabVIEW. For output measurements at external resistance, the current output is recorded by connecting the electrometer and resistance box in series while for voltage output evaluation the electrometer and resistance box were set in parallel. The pressure resilience of the solid-state device was evaluated by varying acceleration/deceleration rate of the LinMot from 1 to 300 m s^-1^. All electrical output evaluations of the t-SEH were done in ambient conditions (T = 22 ± 1°C; %RH = 50 ± 3%).

**Supplementary Note 2**

The chemical environment was further elucidated via X-ray Photoelectron Spectroscopy (XPS) analysis, which reveals distinct spectra for various elements, including C 1s, S 2p, N 1s, O 1s, and B 1s (Figure 2f). The C 1s spectrum displays a prominent peak at 284.7 eV, which indicates aliphatic carbon atoms in the polymer backbone. Additional peaks at higher binding energies are associated with carbon atoms bonded to electronegative functional groups, such as the carbonyl carbon (C=O) in the acrylic acid and acrylamide segments and the carbon adjacent to the sulfonic acid group (C-SO_3_H) in the AMPS component. The S 2p spectrum reveals a characteristic doublet peak and the S 2p3/2 component at approximately 168.0 eV, which confirm the presence of sulphur in the sulfonic acid (SO_3_H) functional group from the AMPS monomer. This incorporation is essential for the ionic properties and hydrophilicity of the terpolymer. In the N 1s spectrum, a peak at approximately 399.8 eV corresponds to the nitrogen atoms in the amide (C-N-H) groups of the acrylamide and AMPS components, which confirms the successful copolymerization of these nitrogen-containing monomers. The O 1s spectrum displays multiple peaks, including a significant peak at approximately 531.5 eV, which is associated with oxygen atoms in the carbonyl (C=O) groups of the acrylic acid and acrylamide units. A shoulder at higher binding energies (approximately 533.0 eV) corresponds to oxygen atoms in the sulfonic acid (SO_3_H) groups of the AMPS moiety. Overall, the comprehensive XPS analysis confirms the successful incorporation of AMPS, acrylic acid, and acrylamide into the terpolymer structure.^1^

The ^1^H NMR spectrum of the terpolymer provides essential insights into its chemical structure and composition through distinct peaks that correspond to specific hydrogen environments. The peak at 2.47 ppm is attributed to the methylene protons in the AMPS monomer. The peak at 3.40 ppm corresponds to the hydroxyl and sulfonic acid protons in the AMPS and acrylic acid components. The peak at 1.43 ppm is associated with the methine proton in the AMPS monomer. The peak at 1.19 ppm corresponds to amino protons in the acrylamide monomer. The peak at 2.13 ppm is assigned to the amide protons in both acrylamide and AMPS monomers^2^ (Figure 2g). Distinct peaks that correspond to various hydrogen environments confirm the successful incorporation of the AMPS, acrylic acid, and acrylamide monomers into the final terpolymer structure. Overall, the tercopolymer, which has sufficient floating hydrogens, electrostatic nitrogen, sulphur, and oxygen ends, saturated hydrocarbons, and charge-withdrawing borates, enables the matrix to accumulate a large amount of electrification charges and slowly dissipate them unless there is a conductive end. Therefore, this tercopolymer is named Electragel.


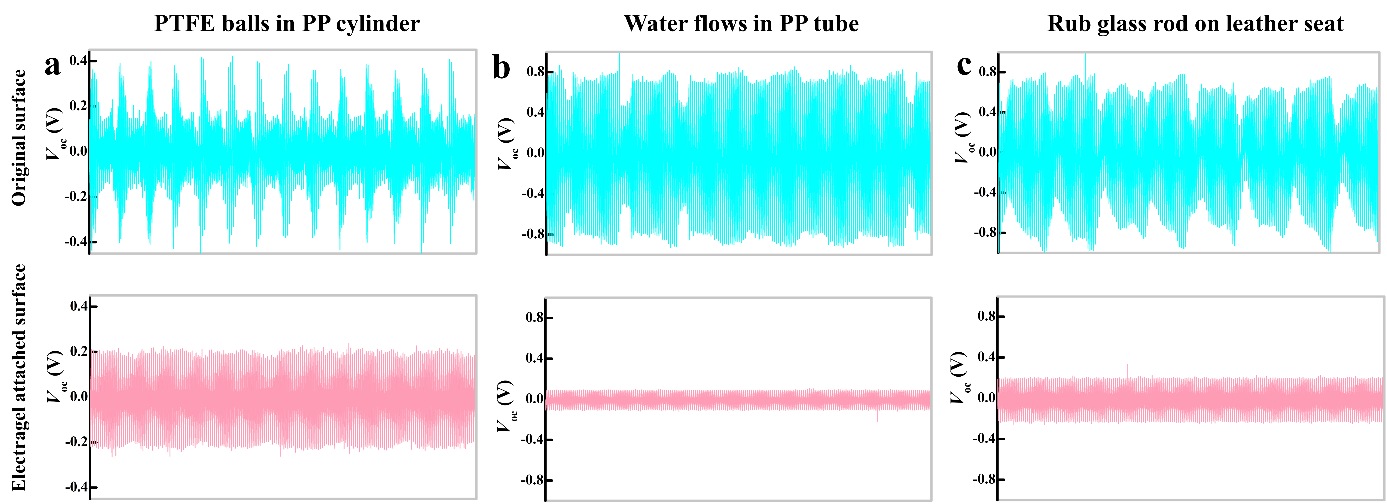


**Figure S1**. Deelectrification dynamics and static charge control by Electragel. Neutralization of static charges by Electragel from (a) PP cylinder with rolling balls, (c) PP tube filled with water, and (d) glass rod rubbed on leather seat.

**Supplementary Note 3**

**Comparative benchmarking of de-electrification performance**

A comparative analysis was conducted to evaluate the static charge dissipation performance of Electragel against conventional hydrogel materials, specifically polyacrylamide (PAAm) and polyvinyl alcohol (PVA). The standard PTFE-ball-in-PET-dish setup (main text Figure 1a) was employed for this evaluation. The open-circuit voltage generated by the rolling balls was measured first for the unscreened system. Subsequently, a 1 cm² patch of each test material was adhered to the dish, and the new stabilized voltage was recorded. As shown in Supplementary Figure S2, the $V_{oc}$ of the control surface without hydrogel displayed significant oscillations with amplitudes up to ±0.6 V, indicating inefficient natural discharge of static buildup. Upon the introduction of Electragel, the voltage profile was dramatically flattened, with peak values restricted below 0.3 V, demonstrating rapid and effective neutralization of accumulated charges. In contrast, PVA hydrogel reduced the amplitude modestly, with peak voltages generally under ±0.4 V, while PAAm hydrogel yielded intermediate suppression to ±0.3 V but did not eliminate the residual oscillations seen in the control. Electragel reduced voltage by 77%, outperforming the other hydrogel materials whose reductions capped at 37%. The results demonstrate a clear performance hierarchy: conventional PAAm and PVA hydrogels provided moderate static dissipation, while Electragel achieved significantly superior voltage reduction. This performance advantage is attributed to Electragel's specialized molecular architecture, which incorporates strongly ionized sulfonic groups (−SO₃⁻) that provide mobile charge carriers, combined with dynamic borate ester crosslinking that facilitates efficient charge neutralization.


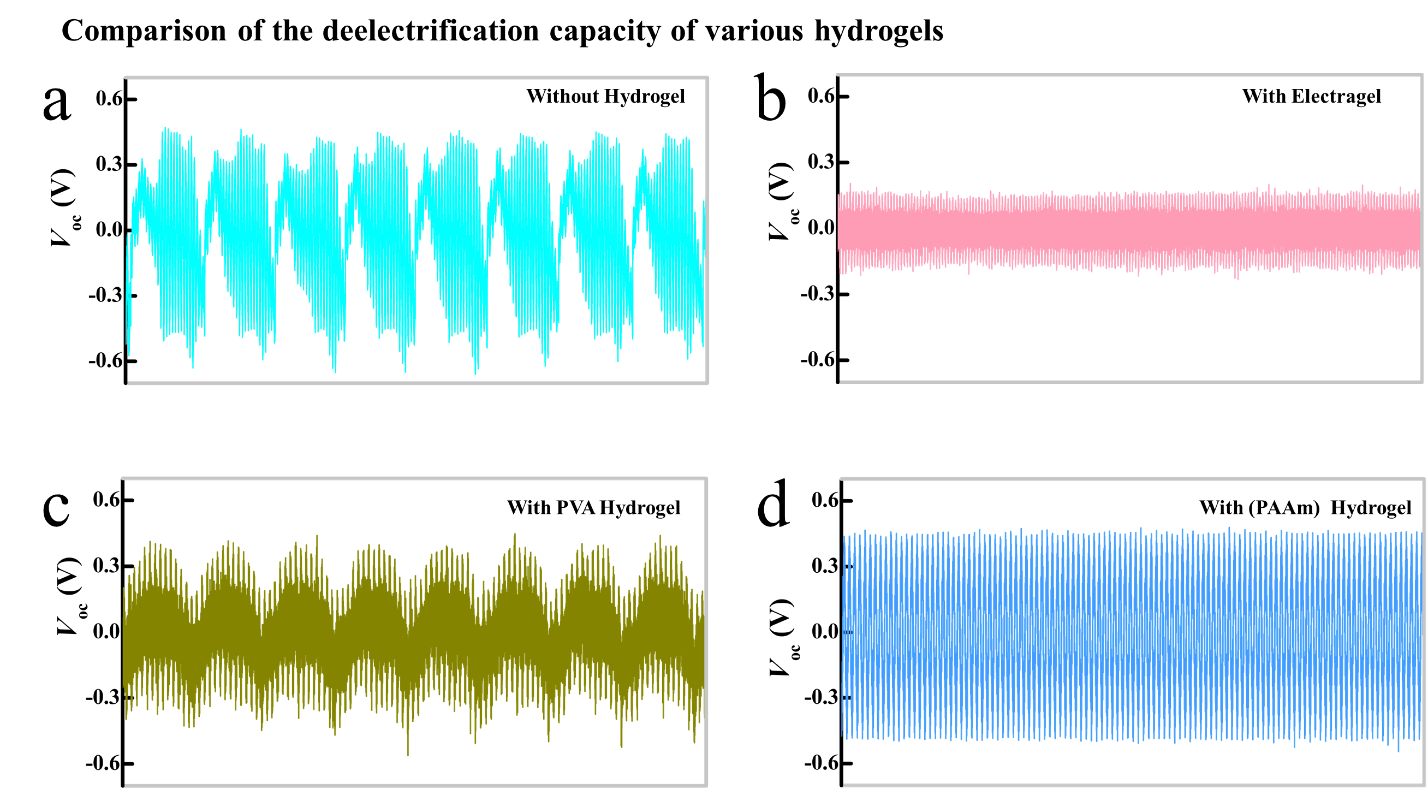


**Figure S2**. Comparative de-electrification performance. Voltage reduction achieved by different 1 cm² hydrogel patches attached to a PET dish: (a) control (no patch), (b) Electragel, (c) PVA hydrogel, and (d) PAAm hydrogel.


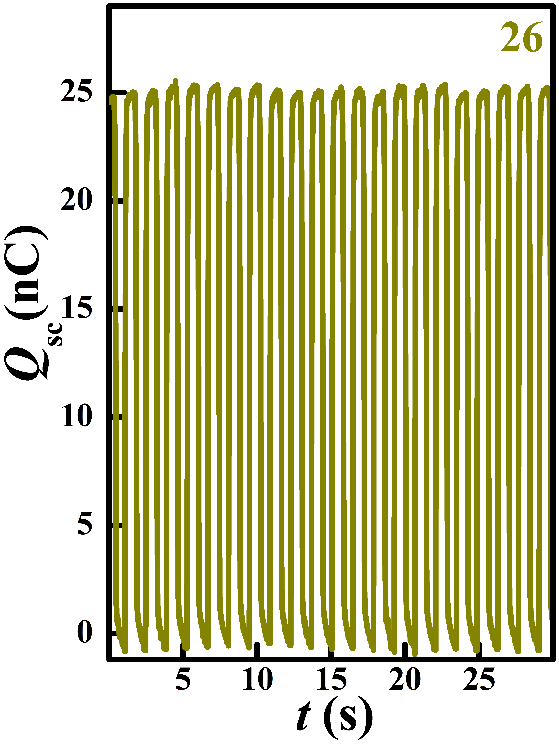


**Figure S3**. Transferred charges from t-SEH with PMMA electrification layer.


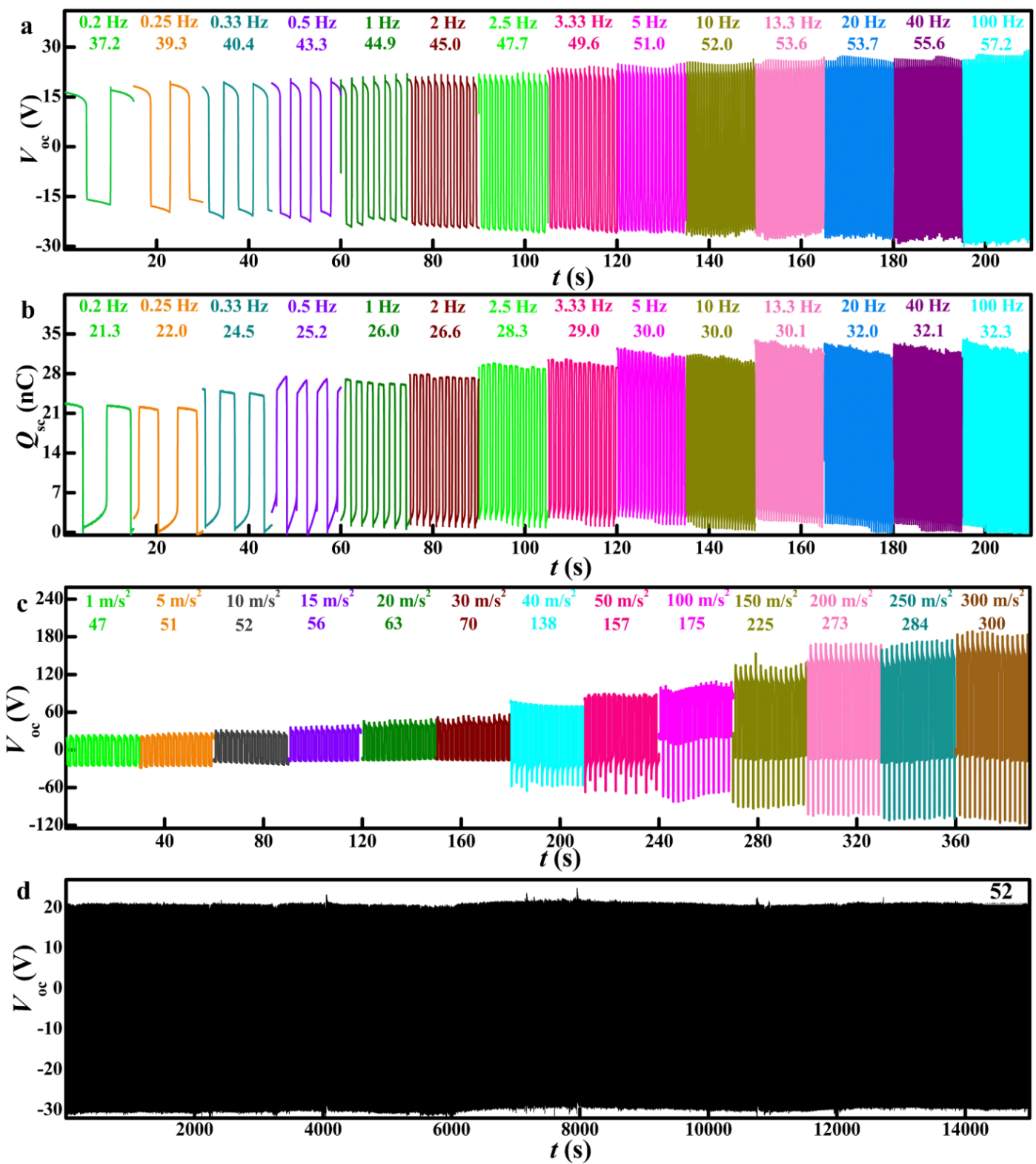


**Figure S4**. Durability of electricity generation via triboelectrification effect. (a, b) The impact of enhanced kinetic energy on device output sensitivity at various frequency ranging from 0.2 Hz to 100 Hz. (c) the impact of the acceleration of contact separating moving counter layer on current output. (d) Stability of output voltage over prolonged contact-separation testing cycles.

**Supplementary Note 4**

**Environmental Stability of Electragel**

The environmental stability of Electragel was rigorously evaluated under controlled humidity and temperature conditions using specialized experimental setups. For humidity testing, experiments were conducted in a closed LinMot aluminium chamber equipped with precision humidity regulators through inlet/outlet ports, enabling controlled variation from 45-90% RH. Temperature variation studies employed a voltage-controlled ceramic heating plate attached to the device substrate, achieving precise temperature control from 5 °C to 40 °C. As shown in Figure S5a, Electragel maintained excellent voltage output across temperatures (90% at 5 °C and 25 °C, 85% at 40 °C), demonstrating minimal thermal degradation. Similarly, Figure S5b shows consistent performance across humidity levels (88-92% efficiency), with only minor variation observed. This remarkable stability stems from Electragel's robust crosslinked network architecture, which maintains structural integrity and ionic conductivity when confined between solid substrates. The sandwiched configuration provides additional stability by limiting environmental exposure while the strong polymer network resists mechanical, thermal, and moisture-induced degradation. Beyond environmental factors, the device demonstrated remarkable robustness to extreme mechanical stress. As shown in Fig. 3f and S4c, the output increased steadily and reliably even when subjected to acceleration/deceleration rates as high as 300 m s⁻², highlighting its mechanical endurance. These results confirm Electragel's reliability for diverse applications, maintaining over 85% performance efficiency across all tested environmental conditions.


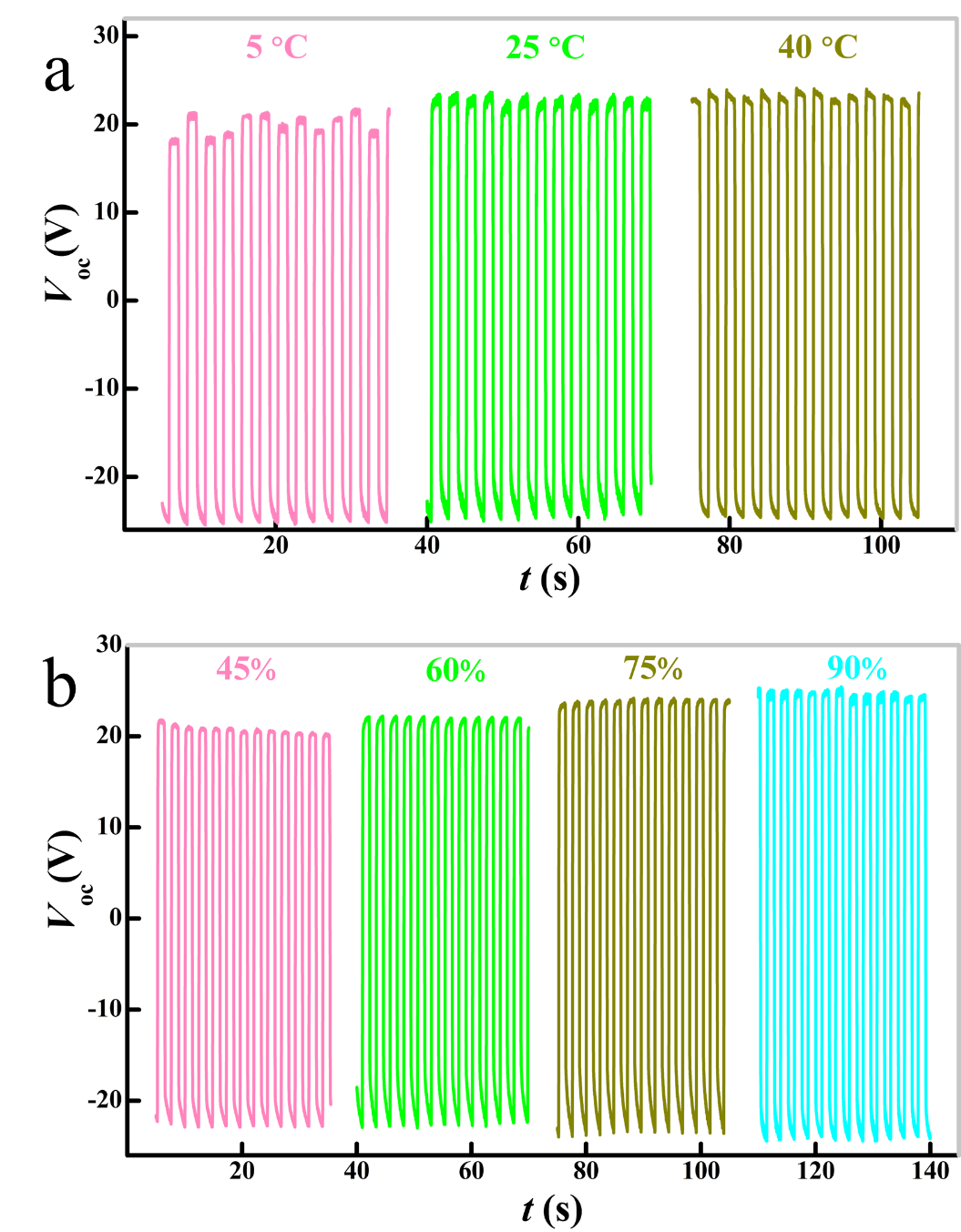


**Figure S5**. Environmental robustness of Electragel. (a) Voltage output remains high across a temperature range of 5 °C to 40 °C. (b) Consistent charge scavenging performance is maintained under varying humidity levels (45-90% RH).


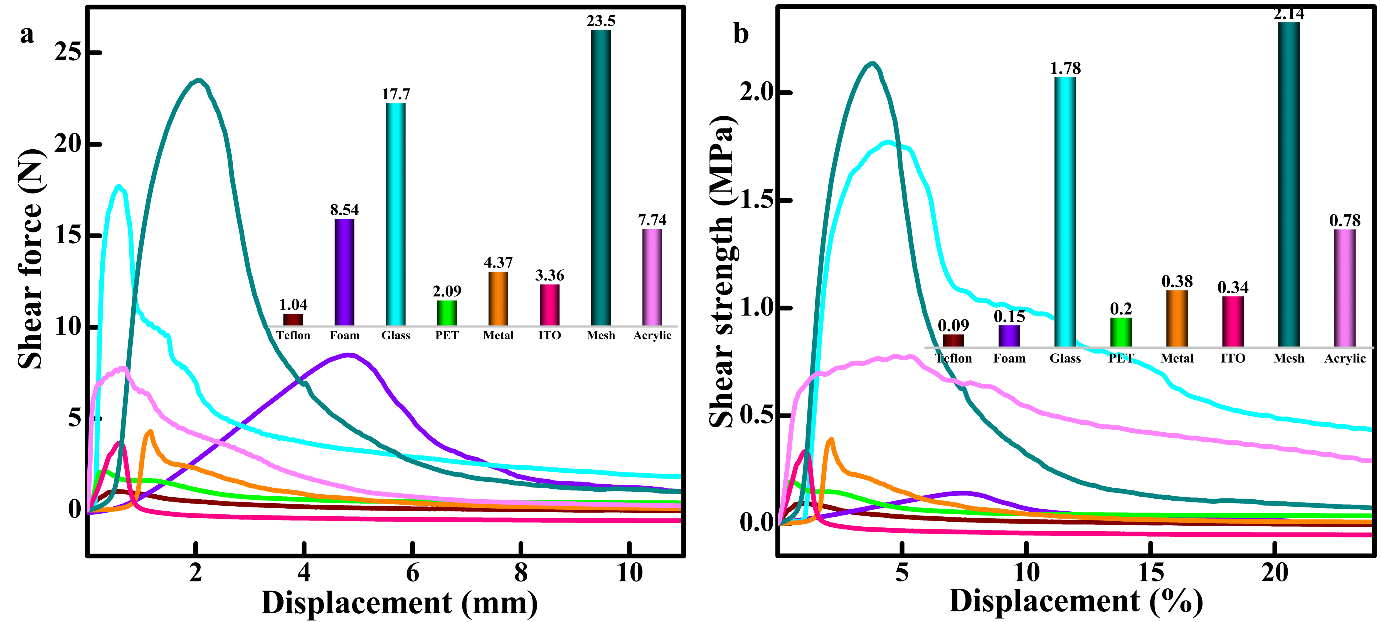


**Figure S6.** lap shear strength profile of Electragel with various tough solids.


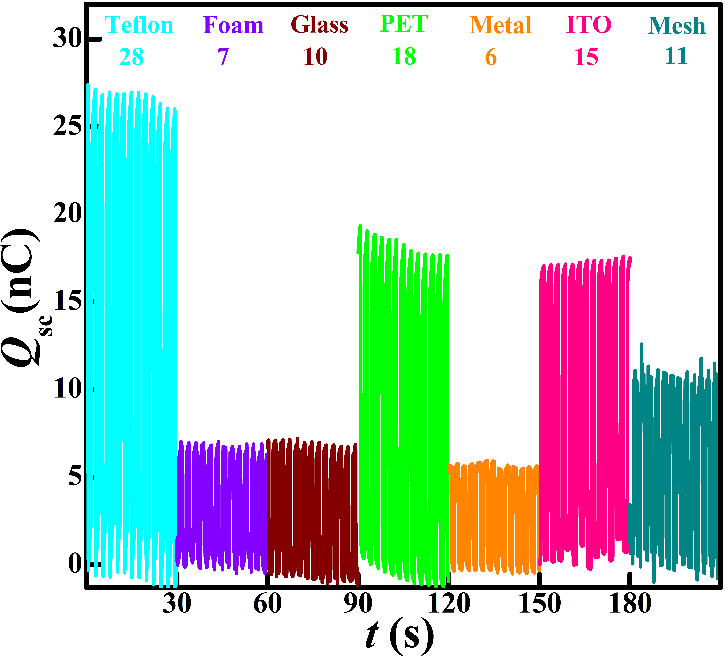


**Figure S7.** Transferred charges from t-SEH with various electrification layers as indicated.


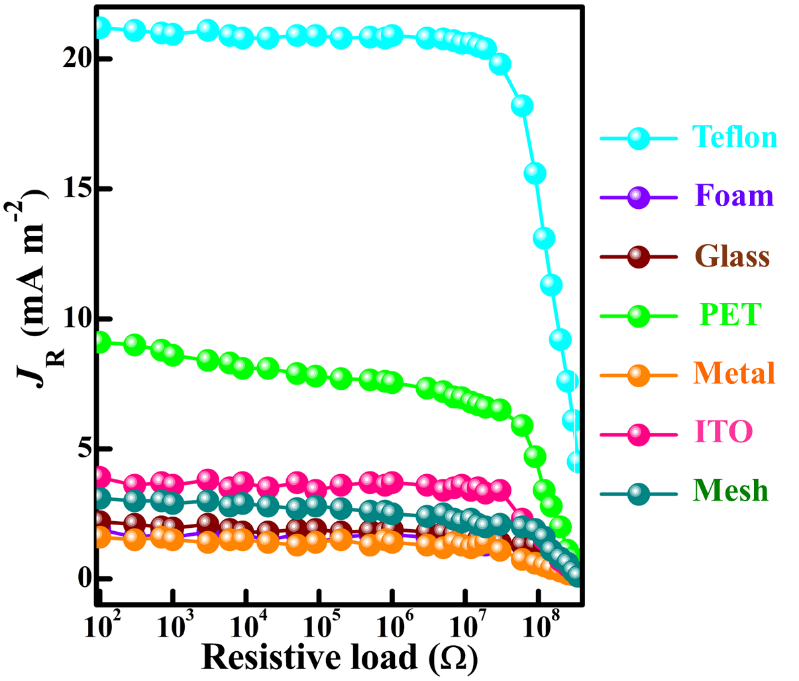


**Figure S8.** Current density profile at external load resistance of t-SEH with various electrification layers as indicated.

**
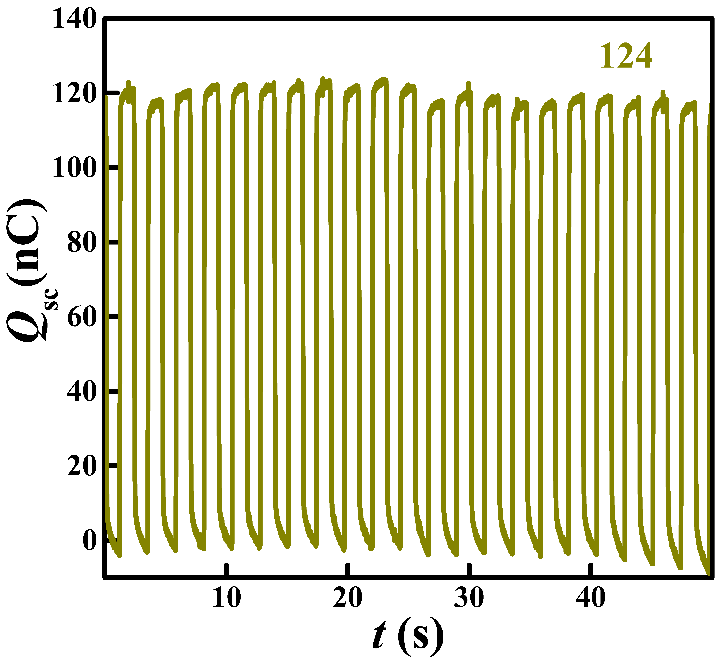
**

**Figure S9.** Transferred charges from t-SEH with elastomer electrification layer.


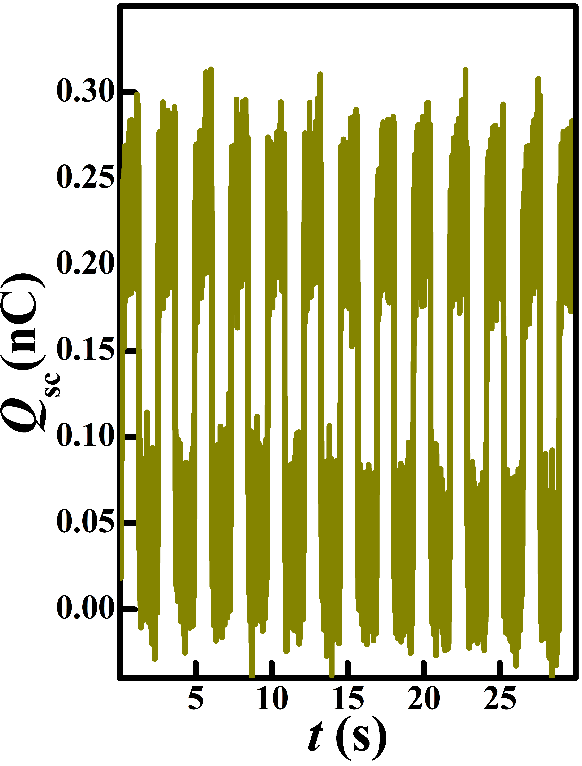


**Figure S10.** Transferred charges from t-SEH with magnetic disc as electrification layer.

**Supplementary Note 5**

**Quantification of magnetic field stability**

To rigorously evaluate Electragel's impact on magnetic field stability and its protective efficacy against demagnetization, we designed a multi-phase experimental protocol comprising three critical tests: (1) Non-Interference Validation: Magnetic flux density of a neodymium disc was measured using a calibrated Gaussmeter (Movie S4) before and after sandwiching Electragel between two magnets, confirming no intrinsic field disruption (54.52 mT pre- vs. 54.52 mT post-placement); (2) Operational Durability Assessment: The magnet was integrated into a t-SEH device and subjected to 500 continuous contact-separation cycles (Movie S5), with flux density measurements pre- and post-cycling revealing exceptional stability with no net losses, as graphically detailed in Figure S11; and (3) Environmental Robustness Testing: Electragel-sandwiched and pristine magnets were exposed to accelerated aging conditions (50 °C/90% RH, 2 hours), where the protected magnet maintained a flux of 54 mT versus the unprotected magnet’s decline to 52 mT (∼5.6% degradation). These results collectively demonstrate that Electragel not only avoids interfering with magnetic fields but actively stabilizes them by scavenging parasitic charges—thereby mitigating eddy current-induced Joule heating—and ensures remarkable retention of magnetic performance under mechanical cycling or environmental stress.


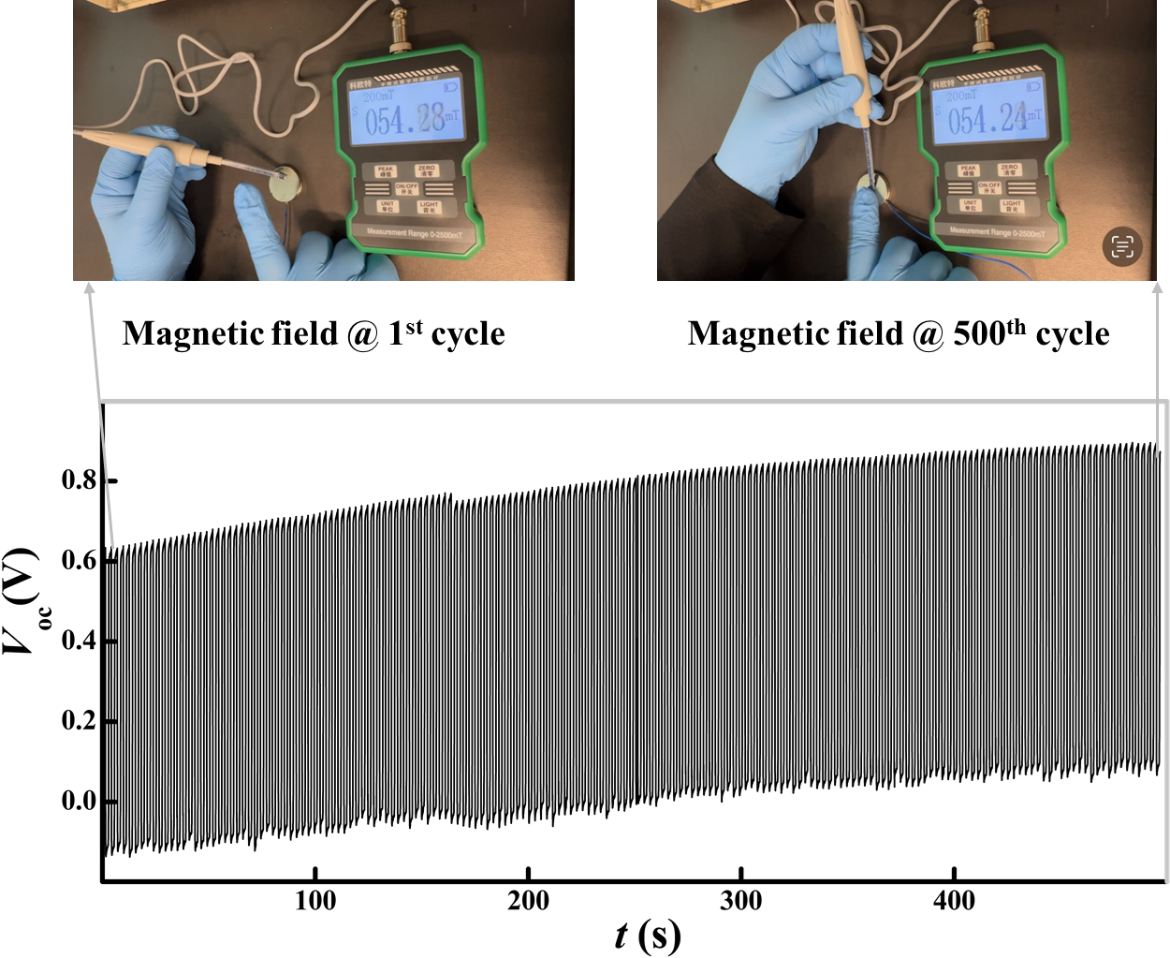


**Figure S11**. Stability of magnetic flux retention before and after 500 mechanical cycles.

**References**

1. J. F. Watts and J. Wolstenholme, *An introduction to surface analysis by XPS and AES*, John Wiley & Sons, 2019.

2. W. Kemp, *Organic spectroscopy*, Bloomsbury Publishing, 2017.

**Other Supplementary materials**

**Movie S1.**

Testing mechanical robustness of Electragel.

**Movie S2.**

Harnessing recoverable energy from Electragel to power LEDs.

**Movie S3.**

Demonstration of wireless power transmission via inductive coupling.

**Movie S4.**

Magnetic field consistency before/after Electragel placement.

**Movie S5.**

Magnetic field stability under mechanical cycling.
